# Simple phasor-based deep neural network for fluorescence lifetime imaging microscopy

**DOI:** 10.1038/s41598-021-03060-x

**Published:** 2021-12-13

**Authors:** Laurent Héliot, Aymeric Leray

**Affiliations:** 1 PhLAM Laboratoire de Physique Des Lasers, Atomes Et Molécules, UMR 8523, CNRS, University of Lille, Lille, France; 2 Laboratoire Interdisciplinaire Carnot de Bourgogne, UMR 6303, CNRS, Université de Bourgogne Franche-Comté, Dijon, France

**Keywords:** Biological fluorescence, Microscopy, Imaging and sensing

## Abstract

Fluorescence lifetime imaging microscopy (FLIM) is a powerful technique to probe the molecular environment of fluorophores. The analysis of FLIM images is usually performed with time consuming fitting methods. For accelerating this analysis, sophisticated deep learning architectures based on convolutional neural networks have been developed for restrained lifetime ranges but they require long training time. In this work, we present a simple neural network formed only with fully connected layers able to analyze fluorescence lifetime images. It is based on the reduction of high dimensional fluorescence intensity temporal decays into four parameters which are the phasor coordinates, the mean and amplitude-weighted lifetimes. This network called Phasor-Net has been applied for a time domain FLIM system excited with an 80 MHz laser repetition frequency, with negligible jitter and afterpulsing. Due to the restricted time interval of 12.5 ns, the training range of the lifetimes was limited between 0.2 and 3.0 ns; and the total photon number was lower than 10^6^, as encountered in live cell imaging. From simulated biexponential decays, we demonstrate that Phasor-Net is more precise and less biased than standard fitting methods. We demonstrate also that this simple architecture gives almost comparable performance than those obtained from more sophisticated networks but with a faster training process (15 min instead of 30 min). We finally apply successfully our method to determine biexponential decays parameters for FLIM experiments in living cells expressing EGFP linked to mCherry and fused to a plasma membrane protein.

## Introduction

Fluorescence lifetime imaging microscopy (FLIM) is a well-established technique for probing the local environment of fluorescent molecules at the nanometer scale. FLIM has thus the unique capability of monitoring changes in temperature, pH, ion (e.g., calcium) concentrations^1^. Because FLIM is also relatively insensitive to fluorophore concentrations, it has been widely used as an efficient and accurate way of quantifying protein conformational changes as well as protein–protein interactions in living cells by detecting fluorescence resonance energy transfer (FRET). The FRET phenomenon is a nonradiative energy transfer from one donor molecule to an acceptor that occurs only when the distance between these two molecules is inferior to 10 nm.

Determination of fluorescence lifetimes can be assessed by two main methods: the frequency domain^2–4^ and the time domain methods^5–7^. In this article we limit our study to this second group. The time-domain (TD) method principle consists in exciting a fluorescent sample with a series of short light pulses and recording the intensity decay histogram *I(t)* emitted by the fluorescent sample. Theoretically, this fluorescence intensity decay decreases exponentially following:

1 $$I\left( t \right) = \mathop \sum \limits_{i = 1}^{\eta } a_{i} \exp \left( { - \frac{t}{{\tau_{i} }}} \right)\;{\text{with:}}\mathop \sum \limits_{i = 1}^{\eta } a_{i} = 1$$

where $\eta$ is the number of species and *a*_*i*_ and τ_*i*_ are respectively the fraction and the lifetime of the species *i*. Proportions and lifetimes values are usually extracted from the FLIM data by fitting experimental decay at each pixel with single or multi-exponential decay models (Eq. 1) using nonlinear least-square algorithms such as the Levenberg–Marquardt, the trust-region-reflective or the Nelder-Meade simplex. These methods are quite efficient for fluorophores exhibiting a single lifetime component because only one parameter has to be determined. In this case, the fluorophore lifetime is equal to the mean lifetime and it can be calculated easily^8,9^. Parameter estimation is more difficult when multiple components are present in the intensity decay, like in FRET experiments where the fluorescence signal emitted by the sample is a mixture of the signal originating from the donor fluorophore alone and from the donor in the presence of the acceptor. The analysis of such samples requires computation time and necessitates a high level of expertise for avoiding local minima leading to potential bias and thus unreliable results^10^. Recently, many efforts have been made to simplify this analysis and to make it accessible to non-expert users by developing for example a non-fitting technique called “phasor”^11–14^. This “phasor” approach converts the intensity decays in two coordinates (*g* and *s*) and thus provide a powerful visualization of data content. However, these 2 phasor coordinates are not enough for resolving biexponential decays constituted with 3 unknown parameters (proportion and lifetimes). In FRET experiments, one fitting parameter has thus to be fixed in order to resolve the other unknown parameters. Usually, this is the donor lifetime value which is fixed from a previous experiment^11,12^, except for biosensing because the donor and acceptor are linked^15^. Few methods have also been proposed to solve the problem without *a-priori* by exploiting multi-frequency lifetime acquisitions^16,17^. However, in order to obtain accurate fitting parameters, they require a high signal to noise ratio (*SNR*) which is not compatible with FLIM experiments of living cells.

Other strategies based on artificial neural network have been proposed for analyzing the temporal decays^18–20^. Because the emitted photons are temporally dependent, the more efficient architectures are based on convolutional networks in one dimension^20^ or three dimensions^19^.

In this work, we propose a simpler neural network based only on dense layers. This simpler architecture can be used because the temporal decays are not directly analyzed but are converted into few physical parameters that will be used as inputs of our neural network. In this work, we use only 4 parameters which are the phasor coordinates (*g* and *s*)^21,22^, the mean and amplitude-weighted lifetimes (τ_m_ and < τ >)^1^. Due to this low number of parameters that are not linearly dependent on each other, convolutional networks are not necessary. By investigating simulations, we demonstrate that our strategy called Phasor-Net is efficient for retrieving all biexponential decays components (lifetimes and proportions). We compare this strategy with standard fitting method based on the minimization of the error estimated from maximum likelihood (MLfit). We demonstrate that Phasor-Net is more precise and less biased than standard fitting approaches for realistic *SNR* simulated data that could be encountered in FRET experiments. We finally apply successfully our method to determine biexponential decays parameters for FLIM experiments in living cells.

### Theoretical framework of Phasor-Net

In order to simplify the artificial neural network, the temporal decays are first converted into four physical parameters that can be calculated easily: the phasor coordinates (*g,s*), the mean lifetime *τ*_*m*_ and the amplitude-weighted lifetime < *τ* > . These parameters are all related to the multiexponential components (see Supplementary Material).

The phasor coordinates are calculated by converting the time domain FLIM data into frequency-domain through a simple Fourier transform of the temporal decay *I(t)*. By separating this complex value into real and imaginary parts, we obtain two quantities noted *g* and *s* which are equivalent to the Fourier sine and cosine transforms of *I(t)*:

2 $$g = \mathop \smallint \limits_{0}^{\infty } I\left( t \right) \times cos\left( {\omega t} \right)dt/\mathop \smallint \limits_{0}^{\infty } I\left( t \right)dt$$

3 $$s = \mathop \smallint \limits_{0}^{\infty } I\left( t \right) \times sin\left( {\omega t} \right)dt/\mathop \smallint \limits_{0}^{\infty } I\left( t \right)dt$$

where ω is the laser repetition angular frequency.

The mean lifetime also called average lifetime corresponds to the first mathematical moment that is defined by:

4 $$\tau_{m} = \mathop \smallint \limits_{0}^{\infty } t \times I\left( t \right)dt/\mathop \smallint \limits_{0}^{\infty } I\left( t \right)dt$$

The amplitude-weighted lifetime < τ > , which is proportional to the area under the decay curve and thus to the steady-state intensity. It corresponds to the normalized total fluorescence signal and it is simply given by:

5 $$\tau = \mathop \smallint \limits_{0}^{\infty } I\left( t \right)dt$$

**Figure 1:**
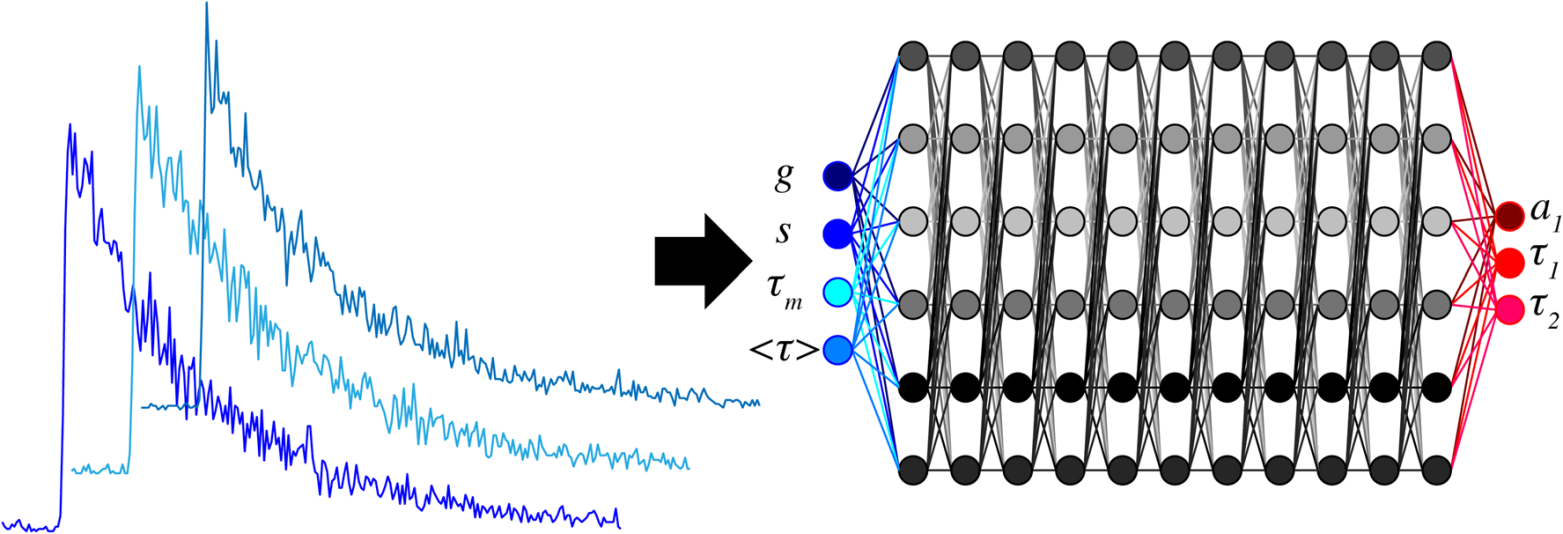
Illustration of the “phasor-based” neural network. The temporal decays are first converted into phasor coordinates noted (*g,s*) mean lifetime τ_m_ and amplitude-weighted lifetime < τ > . These 4 parameters are then used as inputs of a simple neural network composed of 11 dense layers of 6 hidden neurons. The outputs of Phasor-Net are directly the proportion *a*_*1*_ and the lifetimes τ_1_ and τ_2_.

As illustrated in Fig. 1, these parameters are used as inputs of a neural network made only with dense layers. The outputs given by our network are directly the biexponential decays components, namely the proportion *a*_*1*_ and the lifetimes τ_1_ and τ_2_.

## Results

### Optimization of Phasor-Net

First of all, we need to calculate from the training samples, the 4 parameters (*g, s*, τ_m_ and < τ >) used as inputs of our neural network. This calculation takes only 4 ms with a standard laptop (Intel Core i7 CPU, double core at 2.5 GHz). We can then perform the training of the “phasor-based” neural network. For optimizing the Phasor-Net architecture, we have investigated the performance of our dense network with varying number of hidden layers (between 5 and 17).

**Figure 2:**
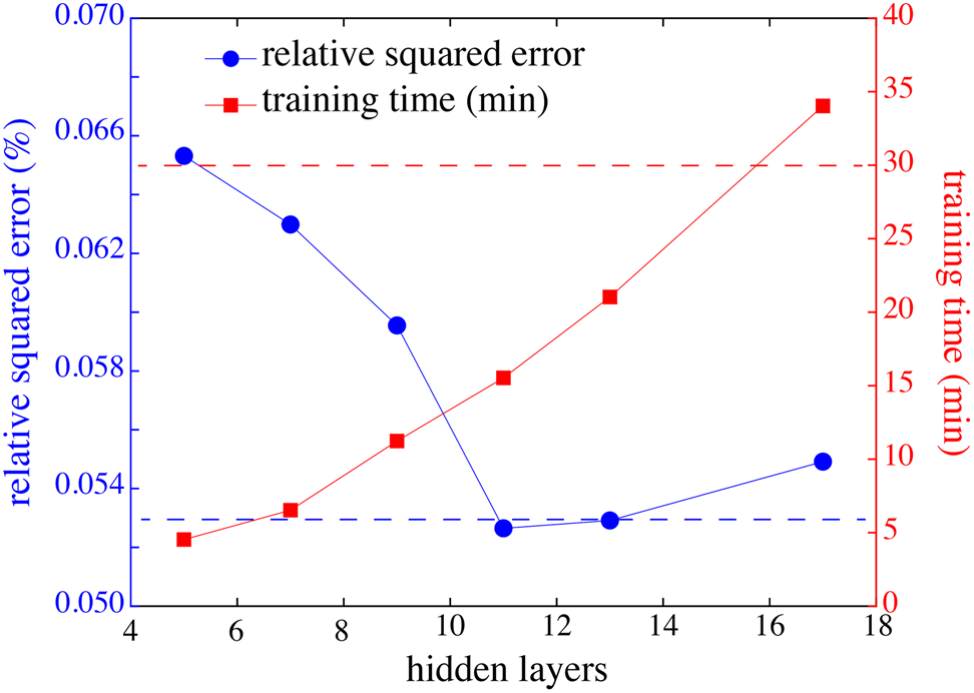
Optimization of Phasor-Net architecture. The relative squared error as a function of the number of hidden layers is shown in blue circles (left y-axis). The corresponding training time is indicated in red squares (right y-axis). The red dotted line corresponds to the fastest training time reported in^20^ and the blue dotted line represents the relative squared error obtained with FLI-Net.

We have represented in Fig. 2 the relative squared error for the validating samples as a function of the number of hidden layers. We found that the relative squared error of the networks constituted with 11 and 13 hidden layers was comparably optimal. The time required for training these different networks are also indicated in Fig. 2. As expected, this training times increases with the number of layers, meaning that eleven hidden layers correspond to a good tradeoff between accuracy and training time.

Thanks to the simplicity of our neural network, the training time of Phasor-Net is thus only 15 min with a standard laptop (Intel Core i7 CPU, double core at 2.5 GHz), which is almost 2 times faster than another existing neural network^20^. Finally, we can note that the relative squared error of our “phasor-based” neural network is comparable with those of FLI-Net^19^.

### Performance evaluation of Phasor-Net on simulations

To evaluate the performances of our “phasor-based” neural network previously trained as described in the Material and Methods section, additional simulated data not included in the training sample have been generated. Numerous factors could influence the performances, such as the signal to noise ratio (*SNR*) or the biexponential decays parameters. All these issues will be discussed in the next sections.

#### Investigation of the effect of the signal to noise ratio

For an optimal FLIM system (with negligible jitter and afterpulsing), signal to noise ratio is the most critical parameter that could influence the performance of the FLIM analysis methods^23^. In this work, we considered 4 distinct *SNR*: 31, 100, 316 and 1000. To mimic FRET experiments, the first and second lifetimes of simulated biexponential decays were fixed respectively to 1.0 ns and of 2.5 ns (corresponding to a FRET efficiency of 60%). We finally considered that these two components are present in equal proportions, meaning that *a*_*1*_ = 0.5.

**Figure 3:**
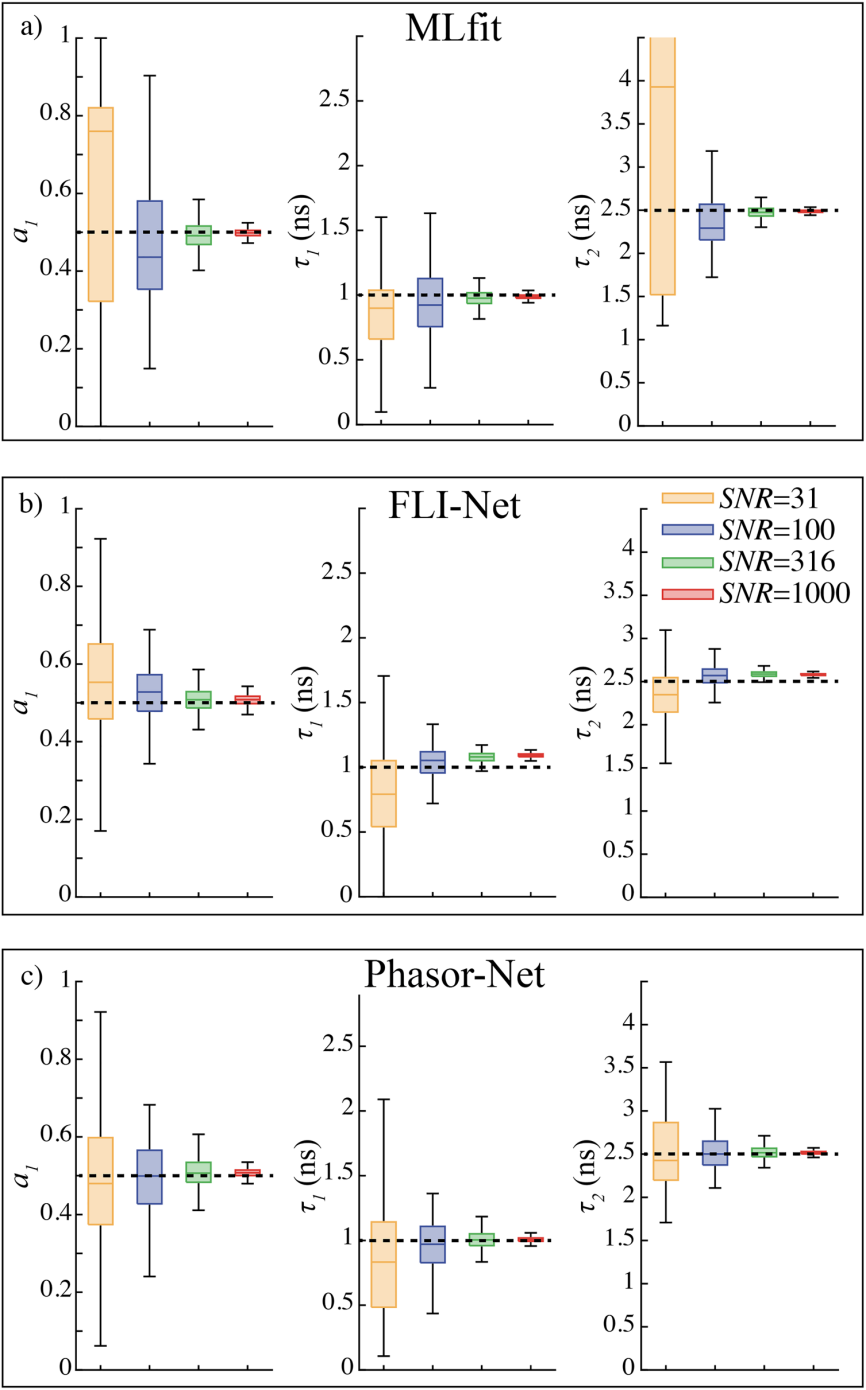
Evaluation of the accuracy of the standard fitting method MLfit (**a**), the convolutional neural network FLI-Net (**b**) and our “phasor-based” neural network Phasor-Net (**c**) for different signal to noise ratio (*SNR*): *SNR* = 31, 100, 316 and 1000. We have investigated three parameters: the proportion a_1_ and the lifetimes τ_1_ and τ_2_. In all graphs, the middle solid line corresponds to the median, the box to the quartile deviations (± 25% of the population around the median) and the black lines to 1.5 times the quartile deviations. We analyzed 1000 simulated decays. The simulated values (a_1_ = 0.5, τ_1_ = 1.0 ns and τ_2_ = 2.5 ns) are indicated with dashed lines.

We have first analyzed these simulated data with standard fitting method because it remains the most usual strategy for determining biexponential component^1,5,24^. The results are reported in Fig. 3a and Table S1. As expected, the dispersion of each parameter (a_1_, τ_1_ and τ_2_) increases when the *SNR* decreases. We can clearly see that MLfit is efficient for analyzing biexponential decays when *SNR* > 100; this is in agreement with previous work concerning the theoretical number of photons required with fitting methods^25^. However, MLfit is not appropriate for low *SNR* data; for instance, it generates errors larger than 50% for *SNR* = 31.

The advantages of deep learning methods compared to MLfit are obvious in Fig. 3. Even if the training *SNR* is fixed, both neural networks (FLI-Net and Phasor-Net) stay accurate for analyzing biexponential decays for a larger *SNR* range (*SNR* = 31, 100, 316 and 1000). We find for instance that the relative errors between the estimated and the true parameters are less than 20% for *SNR* = 31. We note finally that the dispersion of each parameter is reduced for larger *SNR*, as previously reported with MLfit.

In the next section, we will investigate more deeply the performance of these methods for various biexponential parameters.

#### Investigation of the role of the first lifetime

To be as close as possible to FRET experiments, we considered that the donor lifetime is fixed (τ_2_ = 2.5 ns) and that the donor lifetime in presence of the acceptor varies between 0.5 and 2.0 ns, corresponding to a FRET efficiency comprised between 20 and 80%. A *SNR* of 100 was used in order to simulate realistic data.

**Figure 4:**
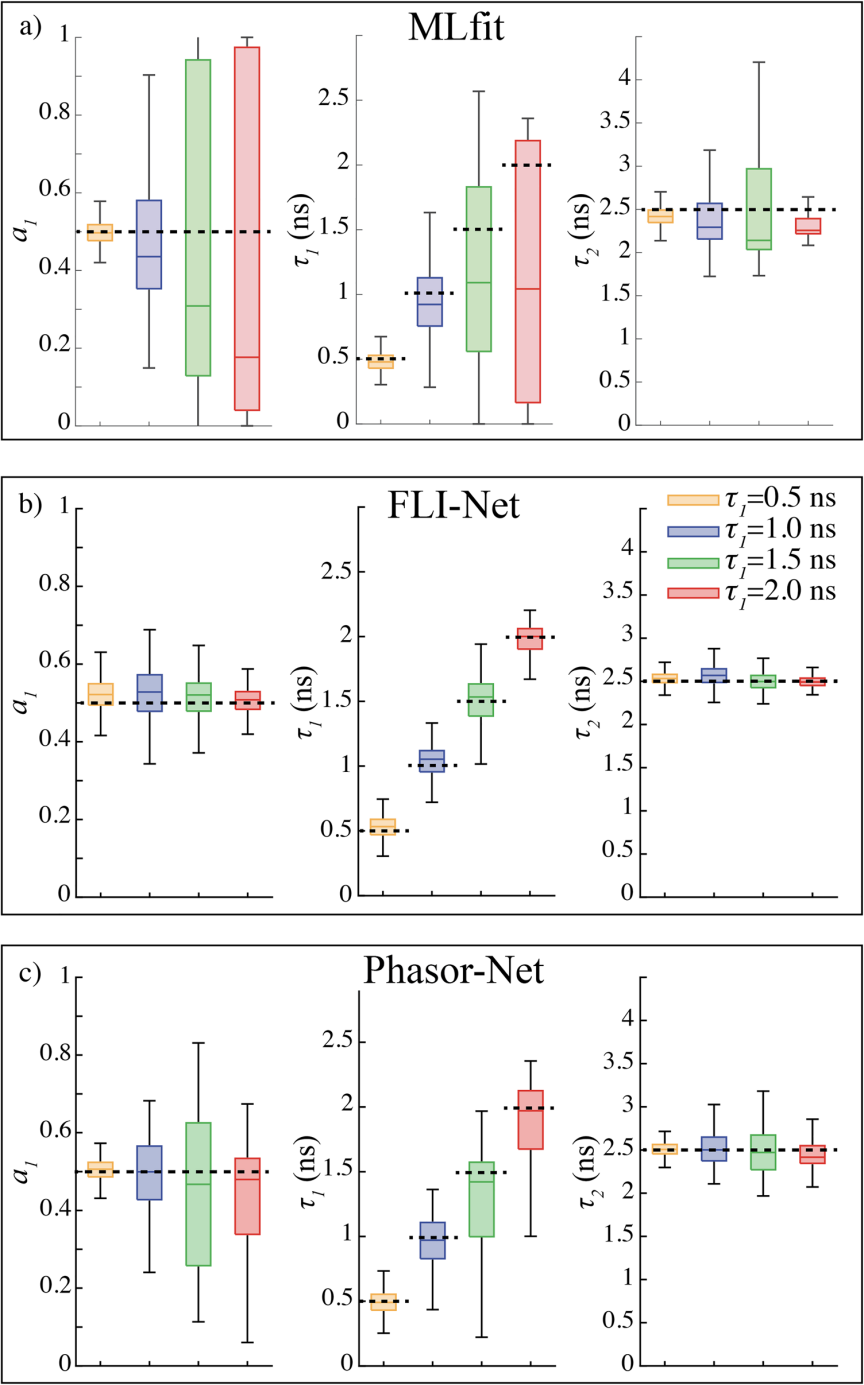
Comparison in parameters accuracy for three methods: (**a**) the standard fitting method MLfit, (**b**) the convolutional neural network FLI-Net and (**c**) the “phasor-based” neural network Phasor-Net. Three parameters are represented: the proportion a_1_ and the lifetimes τ_1_ and τ_2_. The proportion and second lifetime were fixed respectively to 0.5 and 2.5 ns. The first lifetime was varied between 0.5 and 2.0 ns. The simulated values are indicated with dashed lines. In all graphs, the middle solid line corresponds to the median, the box to the quartile deviations (± 25% of the population around the median) and the black lines to 1.5 times the quartile deviations.

As anticipated from our previous results, the analysis of biexponential decays with standard fitting methods is not optimal when the *SNR* is equal to 100. As indicated in Fig. 4a and Table S2, the relative error between the estimated and the true parameters can reach 65%. Furthermore, the estimated parameters are largely dispersed (relative interquartile range larger than 100%) and we clearly see in Fig. 4a that this dispersion increases when the first lifetime becomes higher. This behavior is in agreement with the theoretical work of Köllner and Wolfrum^25^ who demonstrate for a monoexponential decay that the fluorescence lifetime precision of fitting methods Δτ/τ ultimately tends towards the intensity precision Δ*N*/*N* where *N* is the number of photons. In other words, for a constant *N*, the lifetime precision is improved when the fluorescence lifetime decreases.

This theoretical study^25^ is no more applicable for deep learning methods. As indicated in Fig. 4b and Table S2, the relative interquartile range of FLI-Net is lower than 25% and it is does not depend on the value of the first lifetime. For all conditions, FLI-Net is accurate; we report relative errors lower than 7% between the estimated and the true parameters. We obtain similar errors (around 7%) with our “phasor-based” neural network (cf. Fig. 4c and Table S2). However, the dispersion of Phasor-Net is larger than those of FLI-Net. For instance, the relative interquartile range of *a*_*1*_ is 73% for simulated biexponential decays with parameters: *a*_*1*_ = 0.5, τ_1_ = 1.5 ns and τ_2_ = 2.5 ns.

#### Investigation of the role of the proportion ***a***_***1***_

In order to evaluate the performance of fitting and deep learning methods, we have also investigated the effect of the proportion *a*_*1*_ by varying its value between 0.2 and 0.8. As detailed in Sect. 2.a), we have considered a first lifetime of 1.0 ns and a second lifetime of 2.5 ns. We have also kept a *SNR* of 100 for these simulated data which is in usually encountered in FLIM experiments^26^.

**Figure 5:**
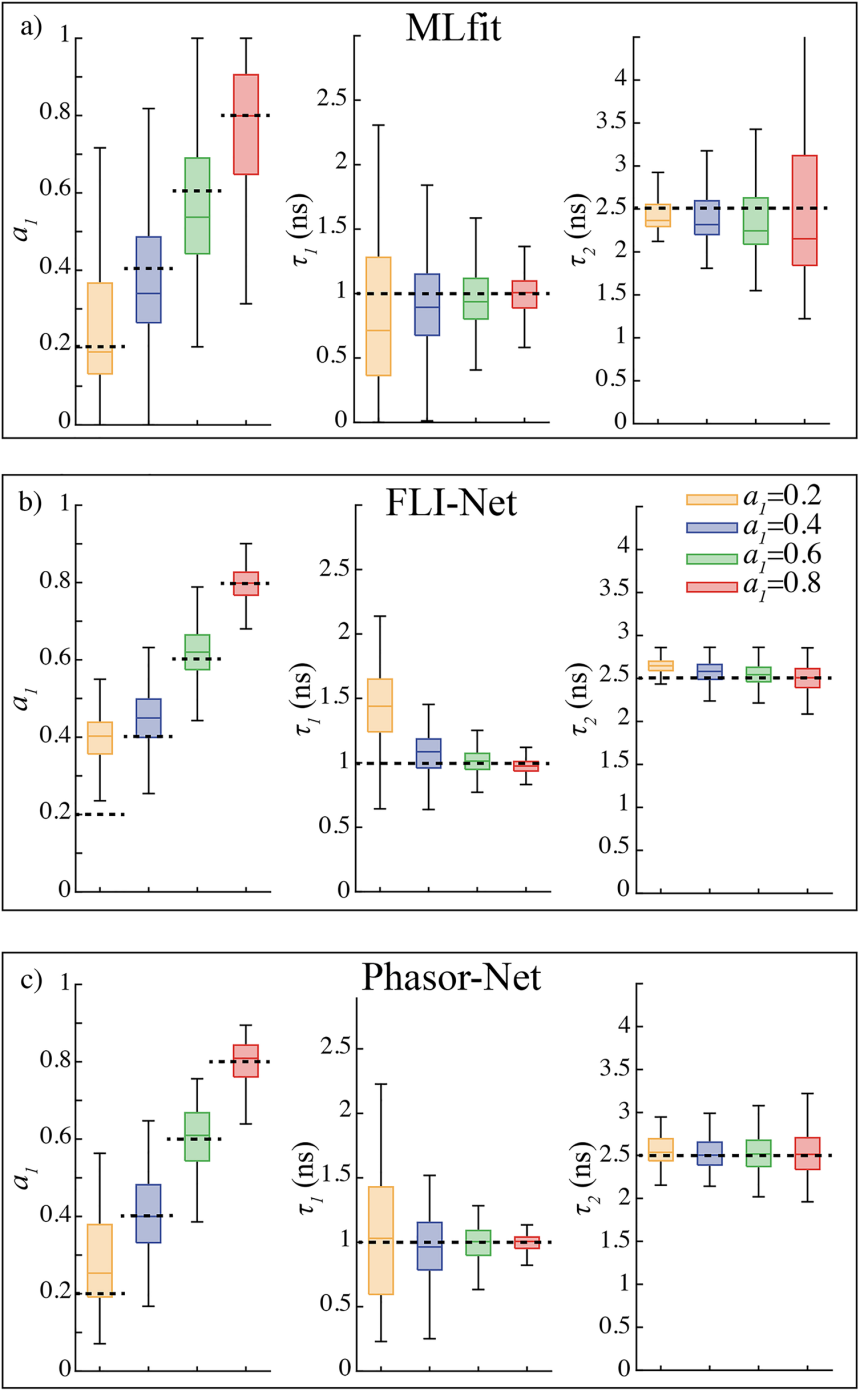
Evaluation of the accuracy in FLIM analysis with three methods: (**a**) the standard fitting method MLfit, (**b**) the convolutional neural network FLI-Net and (**c**) the “phasor-based” neural network Phasor-Net. We have analyzed 1000 simulated histograms whose first and second lifetimes were fixed to τ_1_ = 1.0 ns and τ_2_ = 2.5 ns. We considered 4 distinct proportions: a_1_ = 0.2, 0.4, 0.6 and 0.8. All simulated values are indicated with dashed lines. In all graphs, the middle solid line corresponds to the median, the box to the quartile deviations (± 25% of the population around the median) and the black lines to 1.5 times the quartile deviations.

As shown in Fig. 5a, for such conditions, the standard fitting method gives relatively accurate values with relative errors between estimated and true parameters lower than 30%. The dispersion also is lower than those reported in Fig. 4a but the relative interquartile range can still exceed 100%. Note that this dispersion increases for the second lifetime but decreases for the first lifetime when the proportion becomes larger.

As indicated in Fig. 5b and Table S3, the convolutional neural network FLI-Net gives very low dispersed results (interquartile ranges less than 0.1 for *a*_*1*_ and less than 0.4 ns for the lifetimes). However, we remark also for the first time that the values obtained by FLI-Net can be biased when the proportion is small. Indeed, we report an absolute error as large as 0.2 for the proportion and 0.44 ns for the first lifetime (relative error of 44%).

In comparison with FLI-Net, our “phasor-based” neural network is largely dispersed (we report an interquartile range of 0.2 for *a*_*1*_ and 0.8 ns for the lifetimes) but Phasor-Net gives almost unbiased results. We can see in Fig. 5c and Table S3 that the absolute error for *a*_*1*_ is lower than 0.05 (0.25 instead of 0.20) and for the lifetimes it is less than 0.04 ns (corresponding to a relative error of 4%).

Based on all these simulations, we can deduce that Phasor-Net is a reliable method for retrieving the biexponential decays components. In the next section, we will thus apply it for analyzing experimental data.

### Application on experimental lifetime measurements in vivo

**Figure 6:**
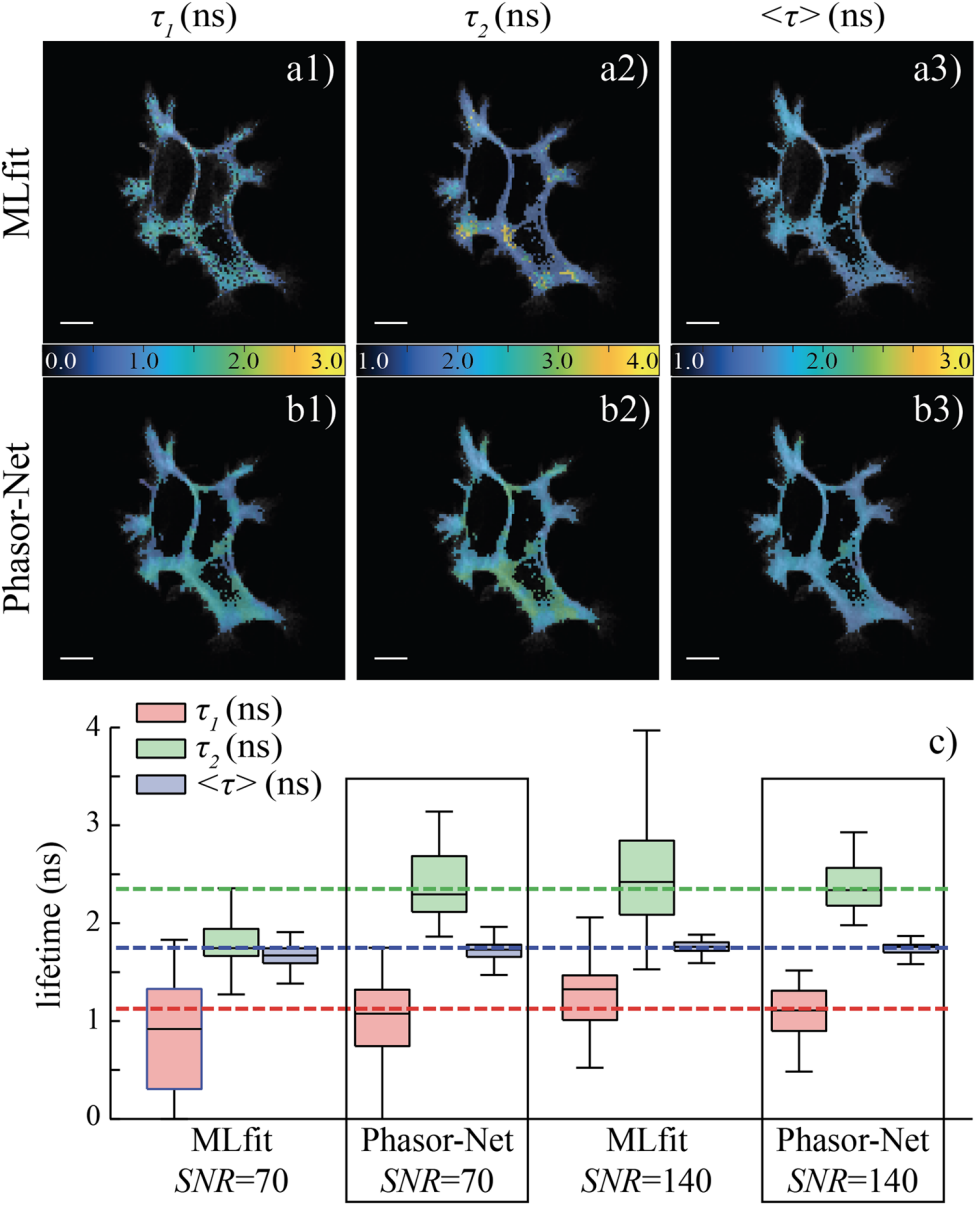
Application of Phasor-Net for FRET measurements in vivo. FRET experiments were performed on HEK293 living cells expressing the EGFP-mcherry tandem linked to a membrane protein. The fluorescence lifetime images obtained from standard fitting method (MLfit) or deduced from “phasor-based” neural network (Phasor-Net) are presented respectively in (**a**) and (**b**). Three FLIM images are shown: (1) first lifetime τ_1_, (2) second lifetime τ_2_, and (3) amplitude-weighted lifetime < τ > (< τ >  = *a*_1_τ_1_ + (1 − a_1_)τ_2_). Scale bar: 10 µm. Respective boxplots are shown in (c). The middle solid line corresponds to the median, the box to the quartile deviations (± 25% of the population around the median) and the black lines to 1.5 times the quartile deviations. The expected lifetimes are also indicated with dashed lines: τ_1_ = 1.16 ns, τ_2_ = 2.35 ns, and < τ >  = 1.75 ns (see text for detail). Phasor-Net is more accurate than MLfit for estimating biexponential decays components.

To demonstrate that Phasor-Net can estimate correctly lifetime components of biexponential decays in FRET experiments, we analyzed FLIM images of live cells constituted with a mean of 5000 photons per pixel (*SNR* of 70). The three lifetime images (τ_1,_ τ_2_ and < τ >) obtained with standard fitting method are shown respectively in Fig. 6a1–a3.

As previously shown and indicated in Fig. 6c, a *SNR* of 70 is not enough to correctly estimate each biexponential decay component with the standard fitting procedure. We report absolute errors of 0.24 ns for τ_1_, 0.6 ns for τ_2_ and 0.08 ns for < τ > . Note that the donor lifetime (τ_2_) was measured to 2.35 ns (+/− 0.13 ns) in another FLIM experiment (data not shown); and the first lifetime was deduced to 1.16 ns (+ /- 0.1 ns) with MLfit by summing all pixels and fixing the second lifetime to 2.35 ns. When we increase the number of photons per pixel up to 20,000 (corresponding to a *SNR* of 140) by applying a binning factor of 10, we reduce notably the absolute errors of lifetimes; they become 0.16 ns for τ_1_, 0.07 ns for τ_2_ and 0.01 ns for < τ > but this strong spatial filtering severely degrades FLIM images.

We have also represented in Fig. 6b1–b3 the three lifetime images obtained with our “phasor-based” neural network. As expected from previous results and illustrated in Fig. 6c, the errors between the estimated and the true lifetimes are small, even for a *SNR* of 70. We report absolute errors of 0.08 ns for τ_1_, 0.05 ns for τ_2_ and 0.02 ns for < τ > , confirming that Phasor-Net is able to accurately estimate all fitting parameters in FRET experiments. By increasing the *SNR* to 140, we reduce again these absolute errors to 0.05 ns for τ_1_, 0.01 ns for τ_2_ and less than 0.01 ns for < τ > . We also reduce the dispersion as illustrated by the smaller interquartile ranges in Fig. 6c.

It is important to insist on the fact that the calculation with Phasor-Net of all lifetime components (a_1_, τ_1_ and τ_2_) was performed in 50 ms with a standard laptop (Intel Core i7 CPU, double core at 2.5 GHz). This computation time is around 3500 times faster than the time required for estimating these parameters with standard fitting method (180 s).

## Discussion

We have demonstrated herein that our “phasor-based” neural network is capable of estimating correctly all biexponential decays parameters (a_1_, τ_1_ and τ_2_) with better precision than those obtained with standard fitting method. However, the bias and the dispersion are not the only important parameters to take into account for characterizing a FLIM image analysis strategy. Once the training process has been performed, the main advantage of the neural network over the standard fitting method (and all the other FLIM image analysis strategies^27–29^) resides in the fact that Phasor-Net does not require any minimization algorithm or fitting procedure for estimating FLIM images but it is laid only on simple mathematical operations. In other words, all biexponential decays parameters calculated in this work have been performed without the necessity to choose the more appropriate algorithm or to fix the value of the donor lifetime^12,30^ or to configure any parameters; in addition, it can be performed on a standard computer. Furthermore, the determination of all biexponential decays parameters for one FLIM image takes only 50 ms (without GPU optimization) which is around 3500 times faster than the computation time with standard fitting method (180 s); confirming that this method can be fully automated and combined with real time high throughput and high content screening for determining both the proportion of interacting proteins and the FRET efficiency^31,32^.

Compared to other existing neural networks that directly use the whole number of temporal channels recorded during TCSPC acquisitions, we reduce in this work the high dimensionality of these temporal histograms by converting them into four parameters: the phasor coordinates, the mean and the amplitude-weighted lifetimes. Due to this low number of parameters that are not linearly related, it is not necessary to apply convolutional neural network but a simple architecture based only on fully connected layers can be used. This dimensionality reduction can be considered as a manual feature extraction and it would be interesting in a future work to investigate computed features extracted from deep learning techniques. However, we demonstrate in this work that our architecture based on simple physical manual features gives almost comparable performance than those obtained from more sophisticated networks^19^. Furthermore, due to its simplicity, the training time of Phasor-Net is around 15 min with a standard laptop (Intel Core i7 CPU, double core at 2.5 GHz), which is faster than other existing networks. To the best of our knowledge, the fastest reported training time was 30 min with an Intel Core i7-4790 CPU processor (quadcore at 3.6 GHz)^20^.

We could imagine that a simpler algorithm such as the k-nearest neighbors would be able to estimate the biexponential components from a set of 4 parameters. In order to evaluate the performance of this approach, we have used the same training samples as Phasor-Net and the simulated data are identical to those detailed in “Resultsb” in section. The results are shown in Fig. S1. After comparison with the results estimated from Phasor-Net that are reported in Figs. 4c) and 5c), we note that the biexponential components are slightly less accurate. For instance, the absolute error for *a*_*1*_ can reach 0.12 and for the lifetimes it can be more than 0.18 ns (compared to 0.05 for *a*_*1*_ and 0.04 ns for lifetimes with Phasor-Net). The simple k-nearest neighbors’ algorithm is thus less precise than our “phasor-based” neural network, probably due to the low *SNR* of our data.

In previous works on convolutional neural networks for lifetime imaging, the training range of the first lifetime was limited to values lower than 1.0 ns (0.4–0.7 ns in^19^ and 0.1–1.0 ns in^20^). Such small values do not correspond to realistic lifetimes in FRET experiments since FRET efficiency is usually lower than 50%^33^ meaning that the first lifetime is larger than 1.2 ns for a usual donor lifetime of 2.5 ns^34^. In this work, we have optimized the training range for FRET experiments by considering a first lifetime comprised between 0.2 and 3.0 ns and demonstrated that our “phasor-based” neural network is valid for various conditions, as illustrated by our simulated and our experimental results.

In this work, the training range of the second lifetime was also comprised between 0.2 and 3.0 ns because the repetition frequency of our excitation laser was 80 MHz corresponding to a time interval of 12.5 ns. Measurement of longer lifetime with this experimental setup is thus not optimal. However, we have investigated the possibility of measuring longer lifetimes with a laser repetition frequency of 20 MHz. We have trained Phasor-Net with a second set of simulated samples whose first and second lifetimes were varying between 0.5 and 15.0 ns. For evaluating the performance of this network, we have fixed the second lifetime to 13.5 ns because it corresponds to the longer donor lifetime reported in^34^. We have considered different proportions (0.2, 0.4, 0.6 and 0.8) and first lifetimes (1.5 ns, 4.5 ns, 7.5 ns and 10.5 ns). The results are shown in Fig. S2. The absolute errors between the estimated and the true parameters in Fig. S2a are 0.04 for proportion and 0.66 ns for lifetimes. They correspond to relative errors of 7% that are comparable with those reported previously. In Fig. S2b, we note an absolute error between estimated and true parameter of 0.06 for proportion and 0.41 ns for lifetimes (relative error of 9%). These errors are slightly larger than those reported previously but are still acceptable. We can thus envision that Phasor-Net could be not limited to FRET experiments but could be applied to all samples emitting fluorescence whose intensity curve exhibits a bi-exponential decay. In practice, there exists a wide range of experimental situations where Phasor-Net could be useful at calculating biexponential decays parameters, such as biosensing or metabolic imaging with autofluorescence measurement^35,36^.

In this work, we have used a Gaussian IRF with FWHM of 32 ps identical to our experimental IRF. It is important to note that the choice of this IRF is not so critical for obtaining correct lifetimes with Phasor-Net but it is well known that it is essential with standard fitting method^29,37^. To illustrate this issue, we have trained Phasor-Net with a Gaussian IRF with FWHM of 32 ps and we have tested it on simulated decays with: (i) a non-Gaussian IRF with the same FWHM; and (ii) a Gaussian IRF whose FWHM was changed to 150 ps. The results are presented respectively in Figs. S3 and S4. No dispersion modifications of the proportion or the lifetimes are visible. We report a slight increase in errors compared to those obtained with a FWHM of 32 ps. For the proportion, the absolute error becomes 0.06 compared to 0.05 and for the lifetimes it is now 0.08 ns compared to 0.04 ns. We can thus conclude that the results obtained with Phasor-Net are still almost unbiased. It can be explained by the fact that the phasor coordinates are almost unchanged when the FWHM is expanded from 32 to 150 ps. Indeed, the convolution of IRF in the temporal domain is equivalent to a modulation decrease and a rotation in the phasor plane depending on the IRF phasor coordinates^20^; and these coordinates are almost not modified when the FWHM is increased from 32 ps (*g* = 1.000; *s* = 0.001) to 150 ps (*g* = 0.999; *s* = 0.034).

Finally, we have applied in this study our “phasor-based” neural network on FLIM images acquired with the well-known TCSPC technique. It would be interesting in a future work to investigate the possibility to apply Phasor-Net to other existing time domain FLIM techniques (based on time gated detector^38,39^ or streak camera^40,41^) and/or to couple with all strategies developed for minimizing the acquisition time (see^42^ for review). In conclusion, because of its simplicity and reliability, Phasor-Net could be of general utility in automating quantitative and real time FLIM measurements.

## Material and methods

### “Phasor-based” neural network (Phasor-Net)

The 4 parameters (*g, s*, τ_m_ and < τ >) are first calculated for each temporal decay. These parameters are then used as inputs of a neural network made only with dense layers. In this work, we have used six hidden neurons which fulfill the fact that the number of hidden neurons should be less than twice the inputs size^43^. For determining the optimal number of hidden layers, we have tested several networks constituted with different numbers of hidden layers ranging between 5 and 17. For each architecture, we have calculated the relative squared error between the targeted and the estimated values.

For using Phasor-net with a specific FLIM system, it is first necessary to generate realistic simulated samples for training (files used for simulations are available here: https://github.com/ayleray/PhasorNet). To mimic experimental conditions, several values are required such as: the number of channels, the temporal resolution of one channel, the laser period, or the FWHM of the IRF. These values should correspond to the experimental parameters of the specific FLIM system (except for IRF as detailed in the discussion section). The training of our neural network was performed with the MATLAB neural network toolbox. Files are available here: https://github.com/ayleray/PhasorNet. We used the Bayesian regularization training algorithm that requires more time but results in good generalization for difficult, or noisy datasets. All hidden layers were activated with a sigmoid function, except the output layer that was activated with a linear function (fitnet)^44^.

Phasor-Net was trained with 50,000 simulated biexponential decays composed with random lifetimes (τ_1_ and τ_2_) ranging between 0.2 and 3 ns and random proportions *a*_*1*_ included between 0.1 and 0.9, covering most of situations encountered in FRET experiments^45^. Because photon emission follows a Poisson distribution, the signal to noise ratio (*SNR*) is equal to the square root of the number of photons. A *SNR* of 316 was applied for these simulations. 15% of these simulated samples were used to validate the training. Due to the stochastic nature of the training, this process was repeated 6 times and only the best training was kept. Because the 4 inputs cover different ranges, they are standardized meaning that they are rescaled in order to have zero mean and unit variance.

### FLIM analysis with convolutional neural network (FLI-Net) and standard fitting method (MLfit)

FLI-Net is a convolutional neural network that has been described by Smith et al.^19^ whose codes are available here: https://github.com/jasontsmith2718/DL4FLI. It is developed with Tensorflow^46^ and the machine-learning library Keras^47^. The training was performed on 300,000 simulated biexponential decays with random lifetimes (τ_1_ and τ_2_) and random proportions *a*_*1*_. We used the same ranges as those applied previously for Phasor-Net: [0.2–3] ns for lifetimes and [0.1–0.9] for *a*_*1*_. The maximal photon count of temporal decays was comprised between 250 and 1000 photons, as described in^19^. Each temporal decay is finally normalized by dividing by its maximal photon count. 15% of these simulations (corresponding to 45,000 samples) were used for validating the training process with a batch size of 20. The mean square error (MSE) was applied as loss function and the RMSprop algorithm with an initial learning rate of 10^–5^ was used as optimizer of this convolutional neural network.

For exhaustive comparison, the biexponential decays components are also resolved with standard fitting method. In this work, we used the Matlab package called “Tcspcfit” that has been developed by Enderlein and Erdmann which is based on an efficient Nelder-Meade simplex algorithm. Codes are available here: https://www.uni-goettingen.de/de/513325.html. We used a convolved autoregressive model for fitting multiexponential decay curves by minimizing the quadratic error estimated from the maximum likelihood (MLfit) because it is more robust than algorithm minimizing the least square deviation (more details can be found in^48^). The initial guess for lifetimes were estimated automatically and they were constrained in the same ranges as those of the training datasets. We have limited the number of iterations to 6000 in order to guarantee that the quadratic error has been minimized correctly and the tolerance was fixed to 10^–12^ for ensuring accurate results.

### Monte Carlo simulations

Simulated biexponential decays were generated on a standard computer by using a Monte Carlo approach, meaning that the arrival time of each photon count is computed from a random number generator whose density probability function is given by the convolution product between the theoretical biexponential decay and the instrumental response function (IRF):

6 $$I\left( t \right) = IRF\left( t \right)*\left( {a_{1} \exp \left( { - \frac{t}{{\tau_{1} }}} \right) + \left( {1 - a_{1} } \right)\exp \left( { - \frac{t}{{\tau_{2} }}} \right)} \right)$$

This process is repeated until the total photon number *N* is reached. To simulate realistic decays, this total photon number follows a Poisson distribution, meaning that the signal to noise ratio is equal to the square root of the total photon counts. In this work, we considered a Gaussian IRF with full width half maximum (FWHM) of 32 ps, as measured by Waharte et al.^6^. To be as close as possible of the experimental conditions, we have considered a laser repetition frequency of 80 MHz, corresponding to a pulse repetition period of 12.5 ns. All simulated biexponential decays are divided into 256 temporal channels.

For generating training samples, we have simulated 300,000 biexponential decays whose proportion *a*_*1*_ was uniformly distributed between 0.1 and 0.9 and whose lifetimes (τ_1_ and τ_2_) were uniformly distributed between 0.2 ns and 3.0 ns. The smallest value was assigned to the first lifetime τ_1_ and the largest to the second lifetime τ_2_. We used a *SNR* of 316 corresponding to a total number of photons of 100,000 to train our “phasor-based” neural network.

In order to investigate the performance of Phasor-Net, we have considered four *SNR*: 31, 100, 316 and 1000 (corresponding respectively to a total of 1000, 10,000, 100,000 and 1,000,000 photon counts) and several biexponential decays components: the proportion varied between 0.2 and 0.8 (0.2, 0.4, 0.5, 0.6 and 0.8), the first lifetime between 0.5 and 2.0 ns (0.5 ns, 1.0 ns, 1.5 ns and 2.0 ns) and the second lifetime was fixed to 2.5 ns, close to the lifetime of EGFP^34^. With these parameters, the FRET efficiency defined by E = 1 − τ_1_/τ_2_^24^ varied between 20 and 80% which is compatible with experimental FRET efficiencies encountered in living cells^33^. For each condition, we have generated 1000 simulated decays, which is large enough to be in good agreement with a Gaussian sampling distribution.

In this work, the relative error between the estimated and the true parameters are calculated with:

7 $$err = \frac{{\left| {X_{m} - X_{t} } \right|}}{{X_{t} }}$$

where X_m_ is the median of the estimated parameter and X_t_ is the expected value.

### FLIM-FRET experiments in living cells

HEK293 cells were cultured in Dulbecco’s modified Eagle’s medium (Invitrogen), supplemented with 10% fetal calf serum and 1% penicillin–streptomycin (Invitrogen), and incubated at 37◦C in a humidified atmosphere of 5% CO2. Cells were plated 12 h before transfection in 32 mm diameter glass coverslips. For obtaining HEK293 cells expressing EGFP (donor) linked to mCherry (acceptor) and fused to a plasma membrane protein, cells were transiently transfected with memb-EGFP-mCherry using FugeneHD (Roche Diagnostic). Details about the construction of the memb-EGFP-mCherry plasmid can be find in^2^. The translated protein is directed to the inner leaflet of the plasma membrane. 24 h after transfection, the culture medium was replaced by L15 medium (Invitrogen) supplemented with 10% fetal calf serum and the observations were performed on a FLIM system based on the time-correlated single-photon counting (TCSPC) technique.

Our TCSPC system was built on a commercial confocal microscope (Leica TCS SP5 X, Leica Microsystems) coupled with a femtosecond mode-locked Ti:Sa laser (Chameleon Ultra 2, Coherent) tuned to the wavelength of 900 nm. HEK293 cells were imaged with a 63 × water immersion objective (NA = 1.2, Leica Microsystems). The resulting epi-collected two-photon excitation fluorescence was selected with a 525/25 bandpass filter (XF3080, Omega Optical) and routed to a high temporal resolution detector (MCP-PMT model R3809U-52, Hamamatsu). The fluorescence intensity decay histograms were divided into 256 channels that were recorded with a dedicated electronic card (SPC 830, Becker & Hickl). The laser power was limited to about 1 mW to avoid photobleaching and/or photodamage effect. The total acquisition time of a FLIM image (composed of 128 × 128 pixels) was 300 s.

Because the signal to noise ratio is lower than 10, we applied a local neighborhood binning. with a factor *n* = 4 corresponding to a surface of (2*n* + 1)^2^ pixels^2^. In order to calculate unbiased phasor coordinates, amplitude-weighted and mean lifetimes (< τ > and τ_m_), the temporal decays have to be background corrected. This value has been estimated from the 100 lowest intensity decays corresponding to non-fluorescent pixels and was subtracted to all temporal histograms. Finally, pixels possessing a total photon number lower than 1240 (corresponding to a *SNR* of 35) have been discarded with standard thresholding. The final FLIM image is then constituted with 1500 pixels whose mean photon number is approximately 5000 photons corresponding to a signal to noise ratio of 70. This simple thresholding selects only fluorescence signal emitted by the desired EGPF fused to a membrane protein and thus suppresses autofluorescence and/or background noise localized everywhere with a lower intensity. As indicated in the supplementary Table S4, when we reduce this threshold, the results are more dispersed and more biased due to autofluorescence and/or background noise.

## Supplementary Information

Supplementary Information.
