# Supplementary material for: Simple phasor-based deep neural network for fluorescence lifetime imaging microscopy
Source: Sci Rep. 2021 Dec 13;11:23858. doi: 10.1038/s41598-021-03060-x (PMC8668934; doi:10.1038/s41598-021-03060-x)
Supplement: Supplementary file 1 — Supplementary Information. [file 41598_2021_3060_MOESM1_ESM.docx]

1. **Relation between the inputs of Phasor-Net and the multiexponential components**

The 4 inputs of Phasor-Net for a given fluorescence decay I(t) are given by the following expressions ^1,2^:

$g={\int_{0}^{\infty} I(t)\times cos\left( \omega t \right)dt}/{\int_{0}^{\infty} I(t)dt}$ (S1)

$s={\int_{0}^{\infty} I(t)\times sin\left( \omega t \right)dt}/{\int_{0}^{\infty} I(t)dt}$ (S2)

$\tau_{m}={\int_{0}^{\infty} t\times I(t)dt}/{\int_{0}^{\infty} I(t)dt}$ (S3)

$\left\langle\tau\right\rangle=\int_{0}^{\infty} I(t)dt$ (S4)

For a multiexponential fluorescence intensity decay with *N* species, *I(t)* is given by:

$I\left( t \right)=\sum_{i=1}^{N} a_{i}\exp\left( -\frac{t}{\tau_{i}} \right)$ with: $\sum_{i=1}^{N} a_{i}=1$ (S5)

The 4 inputs parameters are directly related to these multiexponential components by ^1,2^:

$g=\sum_{i=1}^{N} \frac{f_{i}}{1+\left( \omega\tau_{i} \right)^{2}}$ (S6)

$s=\sum_{i=1}^{N} \frac{f_{i}\omega\tau_{i}}{1+\left( \omega\tau_{i} \right)^{2}}$ (S7)

$\tau_{m}=\sum_{i=1}^{N} f_{i}\tau_{i}$ (S8)

$\left\langle\tau\right\rangle=\sum_{i=1}^{N} a_{i}\tau_{i}$ (S9)

where *f_i_* is the fractional contribution of the fluorescent species *i*, which is given by:

$f_{i}=\frac{a_{i}\tau_{i}}{\sum_{j=1}^{N} a_{j}\tau_{j}}$ (S10)

**References**

1. Digman, M. A., Caiolfa, V. R., Zamai, M. & Gratton, E. The phasor approach to fluorescence lifetime imaging analysis. *Biophys J* 94, L14-6 (2008).

2. Lakowicsz, J. R. *Principles of fluorescence spectroscopy*. (Plenum Publishers, 1999).

1. Supplementary figures


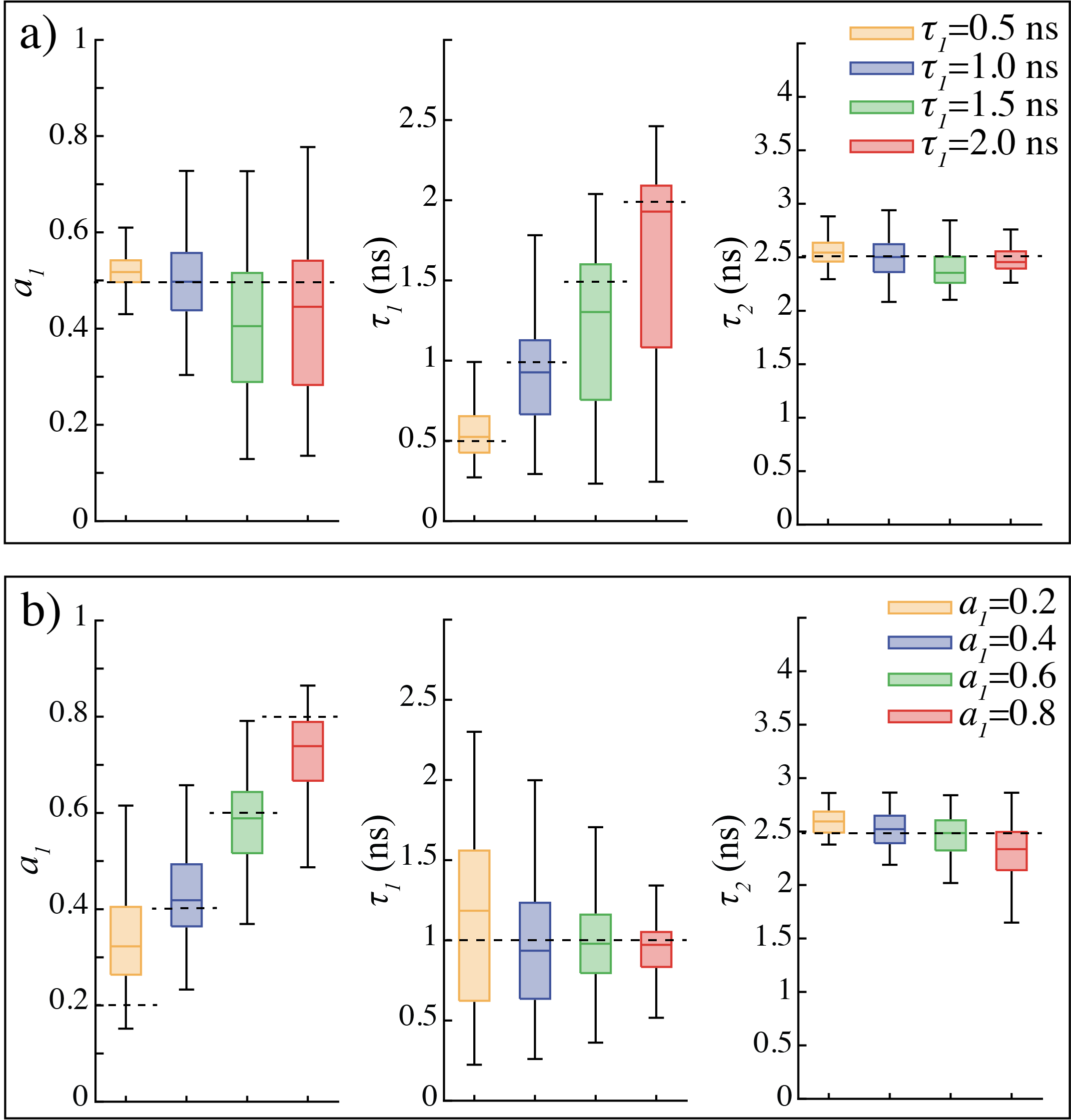


Figure S1: Evaluation of the accuracy of the k-nearest neighbors algorithm for different simulated biexponential decays parameters. (a) The proportion and second lifetime were fixed respectively to 0.5 and 2.5 ns and the first lifetime was varied between 0.5 ns and 2.0 ns. (b) The first and second lifetimes were fixed to 𝜏_1_=1.0 ns and 𝜏_2_=2.5 ns and we considered 4 distinct proportions: a_1_=0.2, 0.4, 0.6 and 0.8. The simulated values are indicated with dashed lines. In all graphs, the middle solid line corresponds to the median, the box to the quartile deviations (± 25% of the population around the median) and the black lines to 1.5 times the quartile deviations. We analyzed 1000 simulated decays with SNR=100. The number of nearest neighbors was 5. We used the KD tree algorithm with Minkowski distance metric and a leaf size of 30.


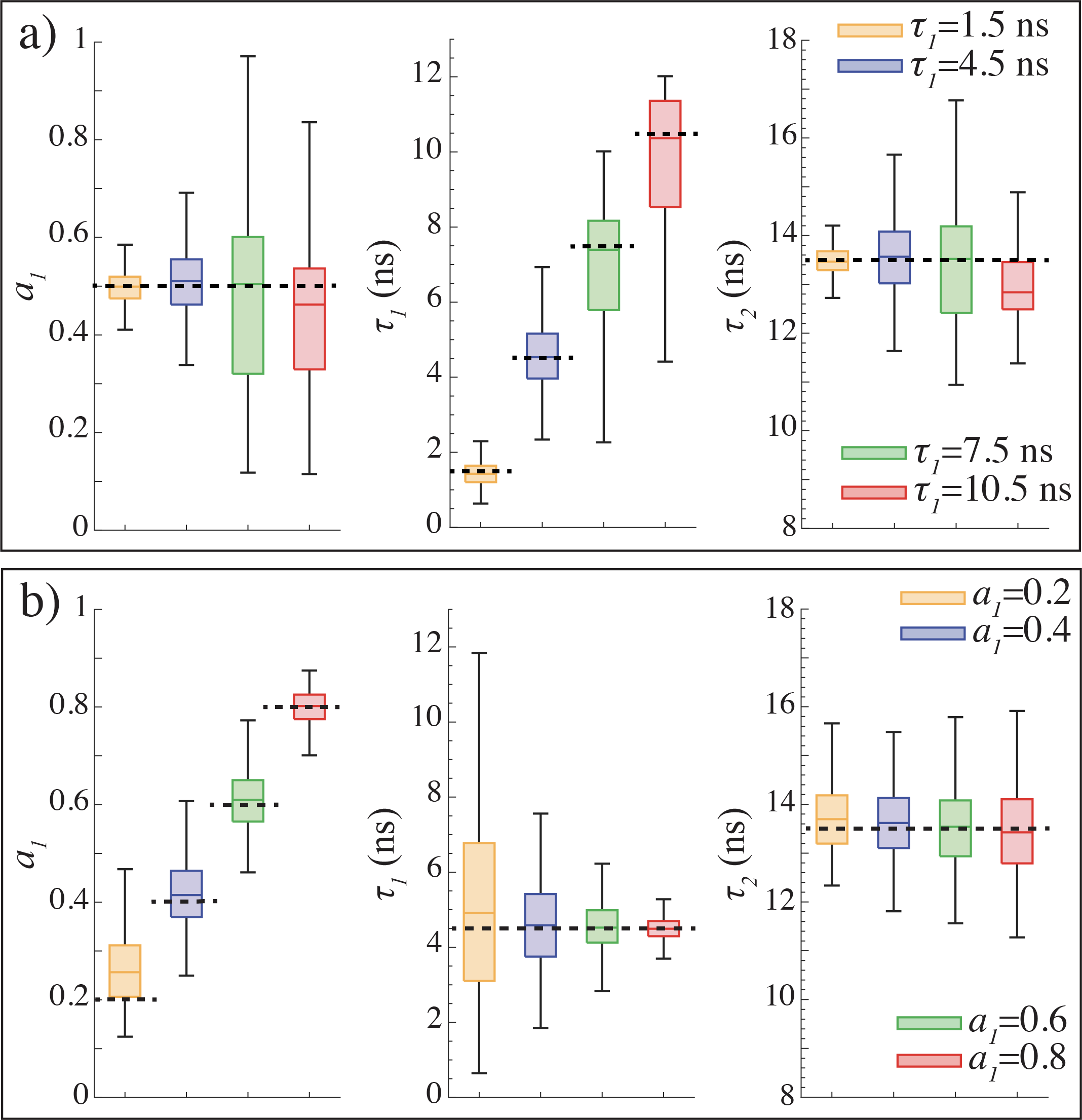


Figure S2 : Evaluation of the accuracy of the “phasor-based” neural network Phasor-Net for longer lifetimes. (a) The proportion and second lifetime were fixed respectively to 0.5 and 13.5 ns and the first lifetime was varied between 1.5 ns and 10.5 ns. (b) The first and second lifetimes were fixed to 𝜏_1_=4.5 ns and 𝜏_2_=13.5 ns and we considered 4 distinct proportions: a_1_=0.2, 0.4, 0.6 and 0.8. The simulated values are indicated with dashed lines. In all graphs, the middle solid line corresponds to the median, the box to the quartile deviations (± 25% of the population around the median) and the black lines to 1.5 times the quartile deviations. We analyzed 1000 simulated decays with SNR=100; the FWHM of the IRF was 32 ps.


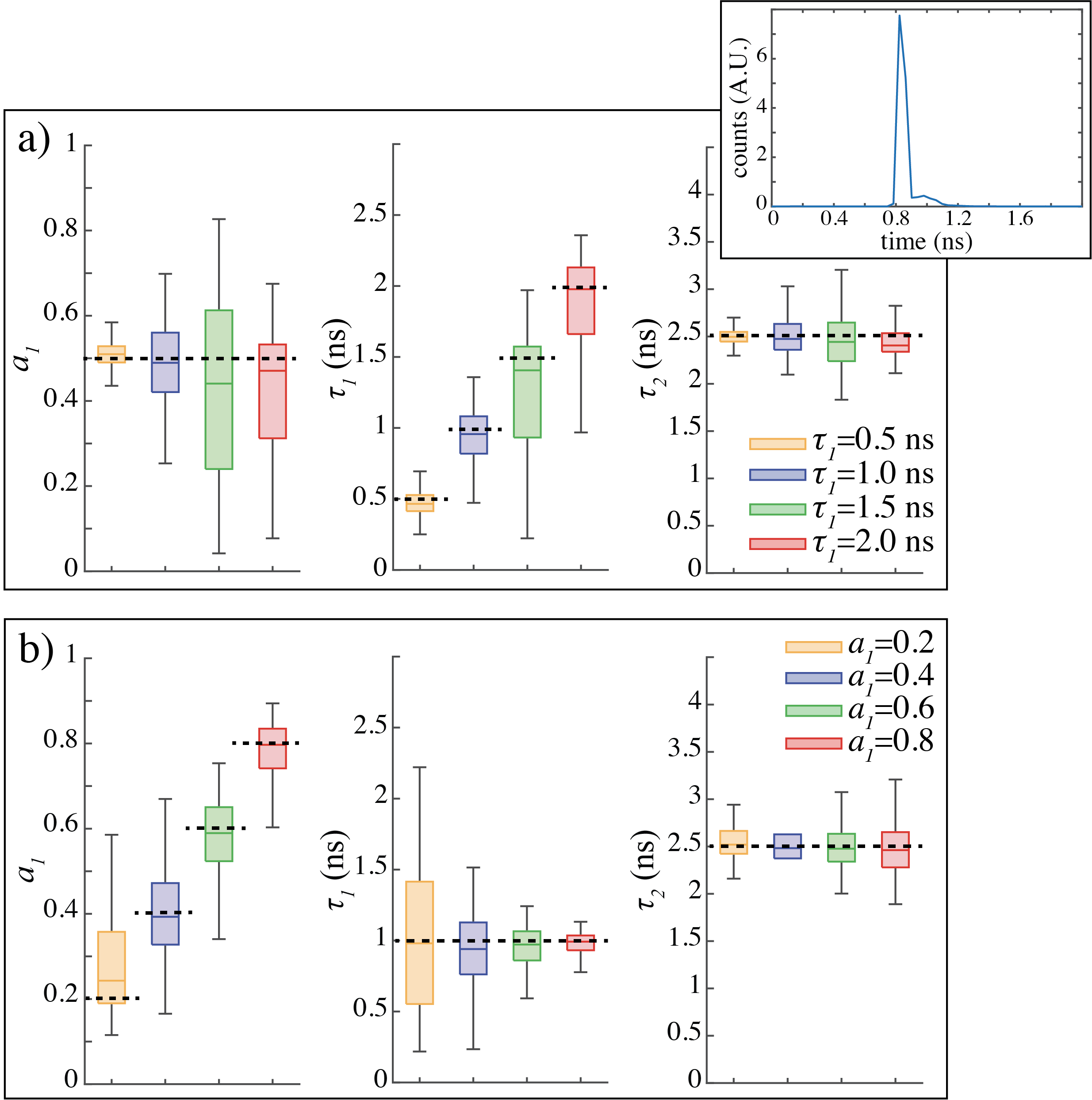


Figure S3 : Evaluation of the accuracy of the “phasor-based” neural network for simulated biexponential decays with a non-Gaussian IRF (shown in the inset). (a) The proportion and second lifetime were fixed respectively to 0.5 and 2.5 ns and the first lifetime was varied between 0.5 ns and 2.0 ns. (b) The first and second lifetimes were fixed to 𝜏_1_=1.0 ns and 𝜏_2_=2.5 ns and we considered 4 distinct proportions: a_1_=0.2, 0.4, 0.6 and 0.8. The simulated values are indicated with dashed lines. In all graphs, the middle solid line corresponds to the median, the box to the quartile deviations (± 25% of the population around the median) and the black lines to 1.5 times the quartile deviations. We analyzed 1000 simulated decays with SNR=100.


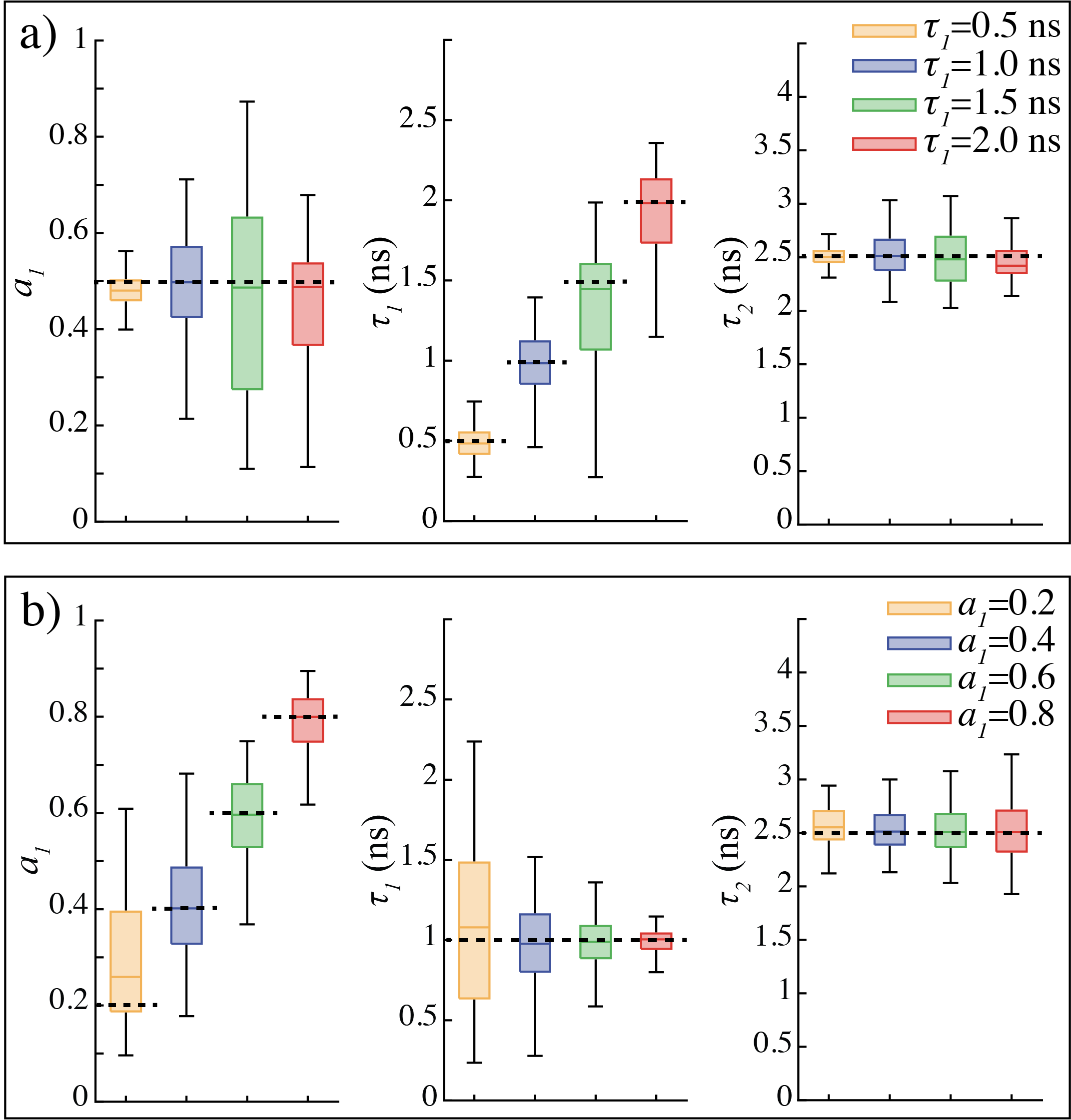


Figure S4 : Evaluation of the accuracy of the “phasor-based” neural network Phasor-Net for simulated biexponential decays with a Gaussian IRF with FWHM of 150 ps. (a) The proportion and second lifetime were fixed respectively to 0.5 and 2.5 ns and the first lifetime was varied between 0.5 ns and 2.0 ns. (b) The first and second lifetimes were fixed to 𝜏_1_=1.0 ns and 𝜏_2_=2.5 ns and we considered 4 distinct proportions: a_1_=0.2, 0.4, 0.6 and 0.8. The simulated values are indicated with dashed lines. In all graphs, the middle solid line corresponds to the median, the box to the quartile deviations (± 25% of the population around the median) and the black lines to 1.5 times the quartile deviations. We analyzed 1000 simulated decays with SNR=100.

1. Supplementary Tables

Table S1: Statistics parameters (median, relative error, interquartile range and relative interquartile range) corresponding to Figure 3

|  | True value (X_t_) | Median (Xm) | | | Error: \|Xm-Xt\|/Xt (%) | | | Interquartile range (iqr) | | | iqr/X_t_ (%) | | |
| --- | --- | --- | --- | --- | --- | --- | --- | --- | --- | --- | --- | --- | --- |
|  |  | ML-fit | FLI-Net | Phasor-Net | ML-fit | FLI-Net | Phasor-Net | ML-fit | FLI-Net | Phasor-Net | ML-fit | FLI-Net | Phasor-Net |
| SNR=31 | |  |  |  |  |  |  |  |  |  |  |  |  |
| a_1_ | 0.5 | 0.76 | 0.55 | 0.48 | 0.52 | 0.11 | 0.04 | 0.50 | 0.19 | 0.22 | 1.00 | 0.39 | 0.45 |
| 𝜏_1_ (ns) | 1.0 | 0.90 | 0.79 | 0.83 | 0.10 | 0.21 | 0.17 | 0.38 | 0.51 | 0.66 | 0.38 | 0.51 | 0.66 |
| 𝜏_2_ (ns) | 2.5 | 3.93 | 2.35 | 2.43 | 0.57 | 0.06 | 0.03 | 3.4 | 0.40 | 0.67 | 1.36 | 0.16 | 0.27 |
| SNR=100 | |  |  |  |  |  |  |  |  |  |  |  |  |
| a_1_ | 0.5 | 0.44 | 0.53 | 0.50 | 0.13 | 0.06 | 0.00 | 0.23 | 0.09 | 0.14 | 0.45 | 0.19 | 0.28 |
| 𝜏_1_ (ns) | 1.0 | 0.92 | 1.05 | 0.97 | 0.08 | 0.05 | 0.03 | 0.37 | 0.16 | 0.28 | 0.37 | 0.16 | 0.28 |
| 𝜏_2_ (ns) | 2.5 | 2.29 | 2.57 | 2.50 | 0.08 | 0.03 | 0.00 | 0.41 | 0.16 | 0.27 | 0.16 | 0.06 | 0.11 |
| SNR=316 | |  |  |  |  |  |  |  |  |  |  |  |  |
| a_1_ | 0.5 | 0.49 | 0.51 | 0.51 | 0.02 | 0.02 | 0.01 | 0.05 | 0.04 | 0.05 | 0.09 | 0.08 | 0.10 |
| 𝜏_1_ (ns) | 1.0 | 0.98 | 1.08 | 1.00 | 0.02 | 0.08 | 0.00 | 0.08 | 0.05 | 0.09 | 0.08 | 0.05 | 0.09 |
| 𝜏_2_ (ns) | 2.5 | 2.48 | 2.59 | 2.52 | 0.01 | 0.03 | 0.01 | 0.09 | 0.05 | 0.10 | 0.04 | 0.02 | 0.04 |
| SNR=1000 | |  |  |  |  |  |  |  |  |  |  |  |  |
| a_1_ | 0.5 | 0.50 | 0.51 | 0.51 | 0.00 | 0.02 | 0.02 | 0.01 | 0.02 | 0.01 | 0.03 | 0.04 | 0.03 |
| 𝜏_1_ (ns) | 1.0 | 0.99 | 1.09 | 1.01 | 0.01 | 0.09 | 0.01 | 0.02 | 0.02 | 0.03 | 0.02 | 0.02 | 0.03 |
| 𝜏_2_ (ns) | 2.5 | 2.49 | 2.58 | 2.52 | 0.01 | 0.03 | 0.01 | 0.02 | 0.02 | 0.03 | 0.01 | 0.01 | 0.01 |

Table S2: Statistics parameters (median, relative error, interquartile range and relative interquartile range) corresponding to Figure 4

|  | True value (X_t_) | Median (Xm) | | | Error: \|Xm-Xt\|/Xt (%) | | | Interquartile range (iqr) | | | iqr/X_t_ (%) | | |
| --- | --- | --- | --- | --- | --- | --- | --- | --- | --- | --- | --- | --- | --- |
|  |  | ML-fit | FLI-Net | Phasor-Net | ML-fit | FLI-Net | Phasor-Net | ML-fit | FLI-Net | Phasor-Net | ML-fit | FLI-Net | Phasor-Net |
| a_1_ | 0.5 | 0.50 | 0.52 | 0.51 | 0.00 | 0.04 | 0.01 | 0.04 | 0.05 | 0.04 | 0.08 | 0.11 | 0.07 |
| 𝜏_1_ (ns) | 0.5 | 0.48 | 0.53 | 0.49 | 0.04 | 0.07 | 0.02 | 0.10 | 0.12 | 0.12 | 0.20 | 0.24 | 0.24 |
| 𝜏_2_ (ns) | 2.5 | 2.42 | 2.53 | 2.51 | 0.03 | 0.01 | 0.01 | 0.14 | 0.10 | 0.11 | 0.06 | 0.04 | 0.04 |
| a_1_ | 0.5 | 0.44 | 0.53 | 0.50 | 0.13 | 0.06 | 0.00 | 0.23 | 0.09 | 0.14 | 0.45 | 0.19 | 0.28 |
| 𝜏_1_ (ns) | 1.0 | 0.92 | 1.05 | 0.97 | 0.08 | 0.05 | 0.03 | 0.37 | 0.16 | 0.28 | 0.37 | 0.16 | 0.28 |
| 𝜏_2_ (ns) | 2.5 | 2.29 | 2.57 | 2.50 | 0.08 | 0.03 | 0.00 | 0.41 | 0.16 | 0.27 | 0.16 | 0.06 | 0.11 |
| a_1_ | 0.5 | 0.31 | 0.52 | 0.47 | 0.38 | 0.04 | 0.07 | 0.81 | 0.07 | 0.37 | 1.63 | 0.14 | 0.73 |
| 𝜏_1_ (ns) | 1.5 | 1.09 | 1.53 | 1.42 | 0.27 | 0.02 | 0.05 | 1.27 | 0.25 | 0.57 | 0.85 | 0.17 | 0.38 |
| 𝜏_2_ (ns) | 2.5 | 2.14 | 2.50 | 2.47 | 0.14 | 0.00 | 0.01 | 0.93 | 0.14 | 0.40 | 0.37 | 0.06 | 0.16 |
| a_1_ | 0.5 | 0.18 | 0.51 | 0.48 | 0.65 | 0.02 | 0.04 | 0.93 | 0.05 | 0.20 | 1.87 | 0.09 | 0.39 |
| 𝜏_1_ (ns) | 2.0 | 1.04 | 2.00 | 1.97 | 0.48 | 0.00 | 0.01 | 2.02 | 0.16 | 0.45 | 1.01 | 0.08 | 0.23 |
| 𝜏_2_ (ns) | 2.5 | 2.26 | 2.49 | 2.42 | 0.10 | 0.01 | 0.03 | 0.18 | 0.08 | 0.20 | 0.07 | 0.03 | 0.08 |

Table S3: Statistics parameters (median, relative error, interquartile range and relative interquartile range) extracted from Figure 5

|  | True value (X_t_) | Median (Xm) | | | Error: \|Xm-Xt\|/Xt (%) | | | Interquartile range (iqr) | | | iqr/X_t_ (%) | | |
| --- | --- | --- | --- | --- | --- | --- | --- | --- | --- | --- | --- | --- | --- |
|  |  | ML-fit | FLI-Net | Phasor-Net | ML-fit | FLI-Net | Phasor-Net | ML-fit | FLI-Net | Phasor-Net | ML-fit | FLI-Net | Phasor-Net |
| a_1_ | 0.2 | 0.19 | 0.40 | 0.25 | 0.06 | 1.01 | 0.27 | 0.23 | 0.08 | 0.19 | 1.17 | 0.41 | 0.93 |
| 𝜏_1_ (ns) | 1.0 | 0.71 | 1.44 | 1.03 | 0.29 | 0.44 | 0.03 | 0.92 | 0.41 | 0.83 | 0.92 | 0.41 | 0.83 |
| 𝜏_2_ (ns) | 2.5 | 2.37 | 2.65 | 2.54 | 0.05 | 0.06 | 0.01 | 0.26 | 0.11 | 0.26 | 0.10 | 0.04 | 0.10 |
| a_1_ | 0.4 | 0.34 | 0.45 | 0.40 | 0.15 | 0.12 | 0.00 | 0.22 | 0.10 | 0.15 | 0.55 | 0.25 | 0.37 |
| 𝜏_1_ (ns) | 1.0 | 0.89 | 1.09 | 0.96 | 0.11 | 0.09 | 0.04 | 0.47 | 0.22 | 0.37 | 0.47 | 0.22 | 0.37 |
| 𝜏_2_ (ns) | 2.5 | 2.32 | 2.58 | 2.50 | 0.07 | 0.03 | 0.00 | 0.39 | 0.17 | 0.27 | 0.16 | 0.07 | 0.11 |
| a_1_ | 0.6 | 0.54 | 0.62 | 0.61 | 0.10 | 0.03 | 0.01 | 0.25 | 0.09 | 0.12 | 0.41 | 0.15 | 0.21 |
| 𝜏_1_ (ns) | 1.0 | 0.94 | 1.01 | 1.00 | 0.06 | 0.01 | 0.00 | 0.31 | 0.12 | 0.19 | 0.31 | 0.12 | 0.19 |
| 𝜏_2_ (ns) | 2.5 | 2.25 | 2.54 | 2.52 | 0.10 | 0.02 | 0.01 | 0.54 | 0.17 | 0.30 | 0.22 | 0.07 | 0.12 |
| a_1_ | 0.8 | 0.80 | 0.80 | 0.81 | 0.00 | 0.00 | 0.01 | 0.26 | 0.06 | 0.08 | 0.32 | 0.07 | 0.10 |
| 𝜏_1_ (ns) | 1.0 | 1.01 | 0.98 | 1.01 | 0.01 | 0.02 | 0.01 | 0.21 | 0.07 | 0.09 | 0.21 | 0.07 | 0.09 |
| 𝜏_2_ (ns) | 2.5 | 2.15 | 2.51 | 2.51 | 0.14 | 0.01 | 0.01 | 1.28 | 0.22 | 0.37 | 0.51 | 0.09 | 0.15 |

Table S4: median and interquartile ranges of first lifetime 𝜏_1_, second lifetime 𝜏_2_ and mean lifetime <𝜏>for different threshold values. True lifetimes are also indicated.

|  | True value | median | | Interquartile range | |
| --- | --- | --- | --- | --- | --- |
|  |  | Th>35 | Th>1240 | Th>35 | Th>1240 |
| 𝜏_1_ (ns) | 1.16 | 0.97 | 1.08 | 0.64 | 0.29 |
| 𝜏_2_ (ns) | 2.35 | 2.31 | 2.30 | 0.64 | 0.29 |
| <𝜏> (ns) | 1.75 | 1.71 | 1.73 | 0.19 | 0.06 |
